# Absence of RIPK3 predicts necroptosis resistance in malignant melanoma

**DOI:** 10.1038/cddis.2015.240

**Published:** 2015-09-10

**Authors:** P Geserick, J Wang, R Schilling, S Horn, P A Harris, J Bertin, P J Gough, M Feoktistova, M Leverkus

**Affiliations:** 1 Section of Molecular Dermatology, Department of Dermatology, Venerology and Allergology, Medical Faculty Mannheim, University Heidelberg, Mannheim, Germany; 2 Department for Dermatology and Allergology, University Hospital Aachen, RWTH Aachen, Aachen, Germany; 3 Pattern Recognition Receptor Discovery Performance Unit, Immuno-Inflammation Therapeutic Area, GlaxoSmithKline, Collegeville, PA 19426, USA

## Abstract

Acquired or intrinsic resistance to apoptotic and necroptotic stimuli is considered a major hindrance of therapeutic success in malignant melanoma. Inhibitor of apoptosis proteins (IAPs) are important regulators of apoptotic and necroptotic cell death mediated by numerous cell death signalling platforms. In this report we investigated the impact of IAPs for cell death regulation in malignant melanoma. Suppression of IAPs strongly sensitized a panel of melanoma cells to death ligand-induced cell death, which, surprisingly, was largely mediated by apoptosis, as it was completely rescued by addition of caspase inhibitors. Interestingly, the absence of necroptosis signalling correlated with a lack of receptor-interacting protein kinase-3 (RIPK3) mRNA and protein expression in all cell lines, whereas primary melanocytes and cultured nevus cells strongly expressed RIPK3. Reconstitution of RIPK3, but not a RIPK3-kinase dead mutant in a set of melanoma cell lines overcame CD95L/IAP antagonist-induced necroptosis resistance independent of autocrine tumour necrosis factor secretion. Using specific inhibitors, functional studies revealed that RIPK3-mediated mixed-lineage kinase domain-like protein (MLKL) phosphorylation and necroptosis induction critically required receptor-interacting protein kinase-1 signalling. Furthermore, the inhibitor of mutant BRAF Dabrafenib, but not Vemurafenib, inhibited necroptosis in melanoma cells whenever RIPK3 is present. Our data suggest that loss of RIPK3 in melanoma and selective inhibition of the RIPK3/MLKL axis by BRAF inhibitor Dabrafenib, but not Vemurafenib, is critical to protect from necroptosis. Strategies that allow RIPK3 expression may allow unmasking the necroptotic signalling machinery in melanoma and points to reactivation of this pathway as a treatment option for metastatic melanoma.

Over the past few years, necroptosis has been established as an alternative programmed form of cell death, contrasting caspase-dependent apoptosis. It is now evident that an ordered activation of the receptor-interacting protein kinases-1 and -3 (RIPK1 and RIPK3), and their downstream substrates is mandatory for the execution of necroptosis.^1, 2, 3^ Under caspase-limited conditions, the necroptotic cell signalling machinery is regulated by RIPK1, with the impact of scaffolding function as compared with kinase function still unclear.^1, 4, 5, 6^ RIPK1 interacts with and either autophosphorylates or transphosphorylates RIPK3 (for review, see Cho *et al.*,^1^ Zhang *et al.*,^2^ He *et al.*,^3^ and Vanden Berghe *et al.*^7^). When RIPK1 is active, RIPK3 phosphorylation and activation occurs within the assembled Necrosome (for review, see Remijsen *et al.*^8^) or Ripoptosome.^4, 9, 10^ RIPK3 then phosphorylates the pseudo kinase mixed-lineage kinase domain-like protein (MLKL).^11^ MLKL in its active form allows its oligomerization, membrane accumulation, and complex formation within cellular membranes of the mitochondria^12^ and cell membranes,^13^ and finally results in necroptosis.^14^

The RIPK1/RIPK3/MLKL signalling network acts as a sensor for genotoxic stress^9^ and also has a key role in necroptosis regulation in keratinocyte skin cancer (SCC).^4^ In these epithelial cancers, cellular inhibitors of apoptosis proteins (cIAPs) block both apoptotic and necroptotic cell death.^4, 5^ Both apoptosis and necroptosis can be increasingly initiated by intrinsic or extrinsic stimuli when IAPs are suppressed by IAP antagonist. Extrinsic apoptosis mediated by activation of death receptors (DRs) such as cluster of differentiation 95 (CD95), TRAILR1/R2 or tumour necrosis factor receptor-1 (TNFR1) through ligation of respective death ligands (DLs) such as CD95L, TNF-related apoptosis-inducing ligand (TRAIL), and TNF initiates apoptosis either by direct activation of the caspase cascade (caspase-8/caspase-3) or via the intrinsic cell death signalling machinery regulated by pro-apoptotic members of the Bcl-2 family followed by caspase-3 activation.^15^ Inhibition of caspase-8 within the death-inducing signalling complex or complex II, or within the Ripoptosome can trigger CD95L-mediated,^5^ TRAIL-mediated^16^ or TNF-induced necroptosis.^8, 17^ A role for apoptosis resistance, cancer maintenance, and progression is widely assumed (for review, see Obexer *et al.*^18^), but the pathophysiological inhibitory or propagating function of necroptosis has not formally been demonstrated in cancer.

Metastatic melanoma has an overall poor prognosis but novel therapeutics have revolutionized clinical practice for different subsets of patients. The use of inhibitors of the V600E- or V600K-mutated proto-oncogene serine/threonine protein kinase B-RAF (e.g., Dabrafenib or Vemurafenib) results in suppression of Ras/Raf/mitogen-activated protein kinase pathways and translate into unfortunately transient clinical responses (for review, see Spagnolo *et al.*^19^). The high recrudescence of metastatic melanoma following the treatment with BRAF inhibitors will potentially require combination therapies that activate additional tumour-inhibitory pathways. Combinations such as BRAF inhibitors with mitogen-activated protein/extracellular signal-regulated kinase kinase (MEK) inhibitors have already yielded impressive results^20^ and other combination therapies may further improve clinical outcome.^21^ As BRAF inhibitors target the cell death pathway at best in an indirect manner, we reasoned that necroptosis induction could represent a novel option to improve melanoma therapy. Our investigations demonstrate for the first time that loss of RIPK3 during melanoma development is critical for necroptosis protection. Reactivation of the RIPK1/RIPK3/MLKL signalling machinery by RIPK3 reconstitution allows IAP antagonist/DL-mediated necroptosis in the presence of Vemurafenib, but not Dabrafenib. Here, Dabrafenib blocks necroptosis by interference with RIPK3-mediated MLKL phosphorylation. Therefore, strategies that increase RIPK3 expression in combination with Vemurafenib, but not Dabrafenib, likely represent an attractive strategy to overcome cell death resistance in melanoma.

## Results

### IAP antagonists sensitize malignant melanoma cells to apoptosis, but not to necroptosis

**Figure 1:**
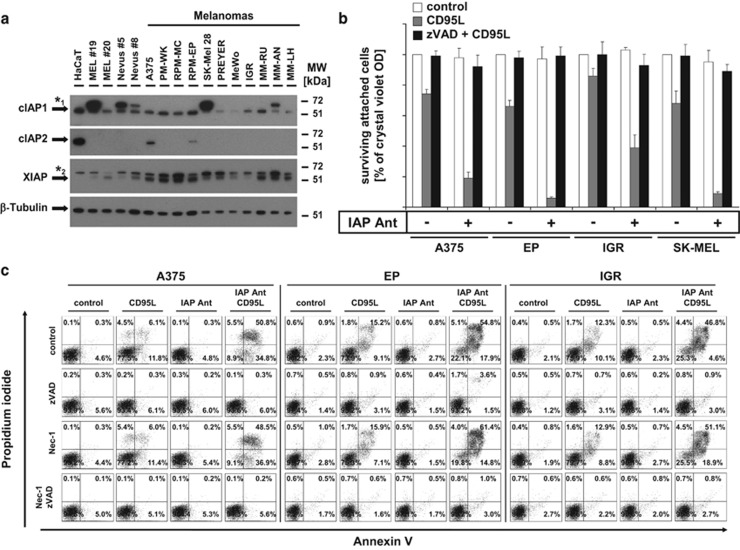
Suppression of IAPs by IAP antagonists in melanomas promotes increased sensitivity to CD95L-induced apoptotic but not necroptotic cell death. (**a**) Heterogeneous expression of cIAP1, cIAP2, and XIAP in HaCaT keratinocytes, primary melanocytes (Mel #19, Mel #20), nevus cells (Nevus #5and #8), and different melanoma cell lines was identified by western blot analysis. Five micrograms of total protein lysates were separated with 4–12% NuPAGE gradient gels before detection of cIAP1 (*1 describes an unspecific band detected with cIAP1 Abs), cIAP2, XIAP (*2 describe an unspecific band recognized by the used XIAP antibody) proteins, and *β*-tubulin as loading control by western blot analysis. One representative experiment of a total of two experiments is shown. (**b** and **c**) IAP inhibition promotes CD95L-induced apoptotic but not necroptotic cell death. (**b**) A375, IGR, SK-MEL and EP melanoma cells were either non-stimulated or pre-stimulated with zVAD-fmk (10 *μ*M) or IAP antagonist (100 nM) alone or in combination for 1 h before stimulation or costimulation with CD95L (0.5 U/ml) for 18–24 h. Surviving attached cells were quantified with crystal violet assay as described in Materials and Methods section. Summary of multiple independently performed experiments (three to four experiments) is shown and S.E.M. was determined accordingly. (**c**) IAP antagonist/CD95L-induced cell death in melanomas is caspase but not RIPK1 kinase dependent. A375, EP, and IGR melanoma cells were either pre-stimulated with DMSO and Ethanol (control), zVAD-fmk (10 *μ*M), Nec-1 (50 *μ*M), or IAP antagonist (100 nM) alone or in respective combinations for 1 h followed by stimulation or costimulation with CD95L (0.5 U/ml) for further 14 h. The externalization of phosphatidylserine as well as the amount of death cells were analysed by AnnexinV/PI double staining as detailed in Materials and Methods. One representative of two independently performed experiments is shown

To investigate the role of IAPs in malignant melanoma, we initially analysed and compared protein expression of different IAPs in cultured primary melanocytes, nevus cells, and malignant melanoma representing different tumour stages and compared them with HaCaT keratinocytes (Figure 1a). cIAP1 and X-linked IAP (XIAP) are expressed in most of the melanoma cell lines examined with lower cIAP1 levels in PREYER, MeWo, IGR, and MM-LH. Low XIAP expression was found in primary melanocytes, MeWo and IGR cells, whereas XIAP is absent in HaCaT keratinocytes as previously described.^22^ In contrast, cIAP2 expression was only detected at low levels in A375 and EP cells, but undetectable in primary melanocytes, nevus cells, and all other melanoma cell lines. Of interest, we detected an additional band reactive with cIAP1 antibody (Ab) in primary melanocytes, nevus cells, as well as in SK-Mel and MM-AH melanoma cells, suggesting that cIAP1 may undergo posttranslational modifications in these cells (Figure 1a). When cIAP1/cIAP2 expression or XIAP function is suppressed by IAP antagonist compound A^23^ (Supplementary Figure 1A), sensitivity to CD95L-mediated cell death is increased. This sensitization was largely independent from the concentrations of IAP antagonist used (Supplementary Figure 1B and C). In addition, low concentrations of IAP antagonists were sufficient for a subtotal decrease of cIAP1 in IGR and also of cIAP2 in A375 cells. This indicates that XIAP is rather more critical as cIAPs for cell death resistance in melanoma cells. In further investigations, we analysed the quality and quantity of IAP antagonist/CD95L-mediated cell death in the presence or absence of a pancaspase inhibitor (zVAD-fmk; Figures 1b and c) in four melanoma cell lines. Under those conditions, melanoma cells were strongly sensitized to DL-mediated caspase-dependent (zVAD-fmk) (Figure 1b, black columns and Figure 1c) but RIPK1-independent (Nec-1), as shown by Annexin-V/propidium iodide (PI) double staining (Figure 1c), apoptosis when compared with cells treated with CD95L alone (Figure 1b, grey columns). Our results demonstrate the indispensable role of IAPs for inhibition of CD95L-mediated apoptotic cell death and suggest that melanoma cells are intrinsically resistant to necroptotic cell death.

### RIPK3 expression is lost during melanoma development

**Figure 2:**
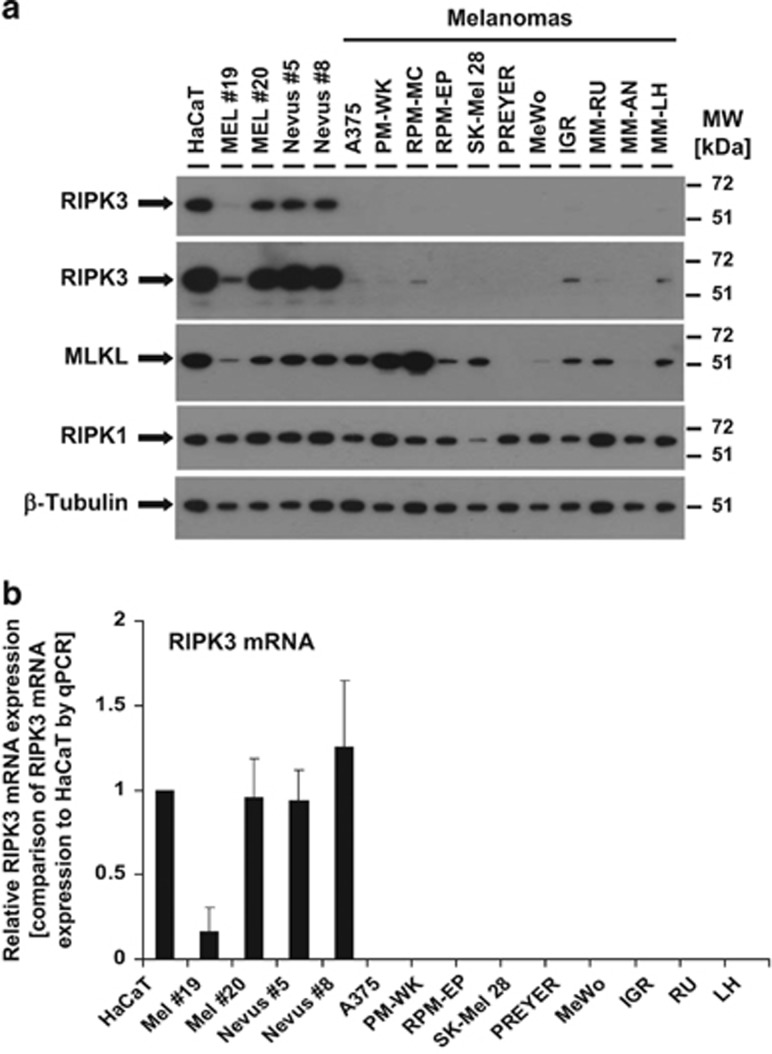
RIPK3 mRNA and protein expression is absent in melanoma. (**a**) Expression of RIPK3, MLKL, RIPK1, and *β*-tubulin as loading control were analysed by western blot analysis from HaCaT keratinocytes, primary melanocytes (Mel #19 and Mel #20), Nevus cells (Nevus #5 and #8), and different melanoma cell lines. Five micrograms of total protein lysates were separated with 4–12% NuPAGE gradient gels before detection of respective proteins by western blot analysis. One representative experiment of a total of two experiments is shown. (**b**) Total mRNA from HaCaT cells, primary melanocytes (Mel #19 and Mel #20), Nevus cells (Nevus #8), and six melanoma cell lines were isolates, reverse transcribed followed by analysis of RIPK3 mRNA expression, as well as housekeeping gene (actin) by quantitative PCR analysis. The mRNA expression of RIPK3 in melanocytes, nevus cells, and melanomas was compared with RIPK3 mRNA expression from HaCaT keratinocytes that serves as control. Summary of two independently performed experiments is shown. The S.E.M. is indicated by error bars

We next reasoned that the lack of necroptosis in melanoma could be a result of a melanoma cell-intrinsic inhibition of proteins relevant for necroptosis induction. We thus examined RIPK1, RIPK3, and MLKL at mRNA and protein levels (Figures 2a and b). When compared with HaCaT keratinocytes that express high levels of RIPK3,^4^ primary melanocytes and nevus cells demonstrated high RIPK3 expression. In marked contrast, expression of RIPK3 is extremely low (A375, MC, IGR, and MM-LH) or fully absent in melanoma cells. MLKL and RIPK1 protein was present in most melanoma cell lines, nevus cells, and primary melanocytes. In addition, PREYER, MeWo, and MM-AN cells showed low or absent expression of MLKL (Figure 2a). To investigate whether the absence of RIPK3 expression was a result of a lack of transcription of RIPK3, we next investigated mRNA expression of RIPK3. When compared with HaCaT keratinocytes, primary melanocytes (Mel #20 but not Mel #19) and both cultured nevus cells highly expressed RIPK3 mRNA. In marked contrast, RIPK3 mRNA expression was absent or below detection level in all melanoma cell lines studied (Figure 2b). Taken together, these observations raised the possibility that the lack of RIPK3 mRNA and protein expression explains the absence of necroptosis in melanoma cells.

### RIPK3 allows for IAP antagonist/CD95L-induced necroptosis in malignant melanoma

**Figure 3:**
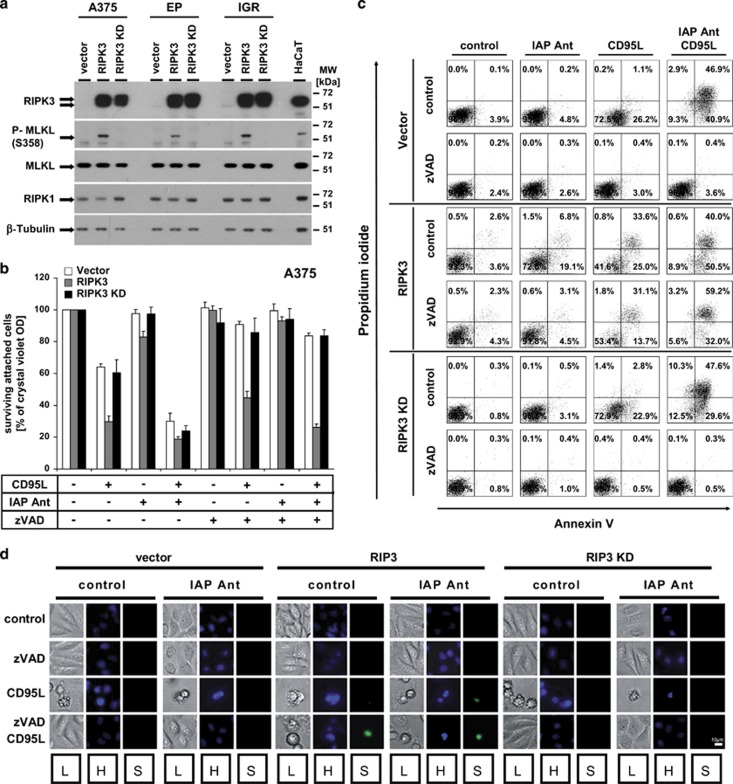
Reconstitution of RIPK3, but not of RIPK3-KD, promotes spontaneous MLKL phosphorylation and necroptotic cell death. (**a**) Overexpression of RIPK3 and RIPK3-KD (D160N) in melanomas. Retroviruses expressing human RIPK3 or RIPK3-KD, or vector controls were used for transduction of A375, RPM-EP, and IGR melanoma cells. Transduced cells were selected and analysed for RIPK3 overexpression, as well as for MLKL, p-MLKL, and *β*-tubulin as loading control. Five micrograms of total protein lysates were separated with 4–12% NuPAGE gradient gels before detection of respective proteins by western blot analysis. One representative experiment of a total of three independent performed experiments is shown. (**b**–**d**) RIPK3 but not RIPK3-KD promotes caspase-independent cell death. (**b**) A375 cells with expression of control (vector), RIPK3, and RIPK3-KD were either non-stimulated or pre-stimulated with zVAD-fmk (10 *μ*M) or IAP antagonist (100 nM) alone or in combination for 1 h before stimulation or costimulation with CD95L (0.5 U/ml) for 18–24 h. Surviving attached cells were quantified with crystal violet assay as described in Materials and Methods. Summary of multiple independent performed experiments (four experiments) including the S.E.M. of all experiments is depicted. (**c**) For phosphatidylserine (PS) externalization and cell death quantification, AnnexinV/PI double stainings was performed. A375 cells with expression of control (vector), RIPK3, and RIPK3-KD were pre-stimulated with DMSO/Ethanol (control), zVAD-fmk (10 *μ*M), or IAP antagonist (100 nM) alone or in respective combination for 1 h before stimulation or costimulation with CD95L (0.5 U/ml) for 14 h. Externalization of PS and cell death were analysed after Annexin V/PI double staining by FACS analysis as described in Materials and Methods section. One of two independent experiments is shown representatively. (**d**) A375 cells with expression of the respective constructs were stimulated as described in **c** followed by qualitative characterization of cell death by fluorescent microscopy after Hoechst/Sytox green double staining as detailed in the Materials and Methods section. One of two independent experiments is shown (L, through Light-microscopy; H, Hoechst staining; S, Sytox green staining)

To functionally investigate the role of RIPK3 in apoptotic and necroptotic cell death in melanoma, we next reconstituted RIPK3 in a number of melanoma cell lines (Figure 3a). Successful RIPK3 overexpression, but not its kinase-inactive mutant D160N (RIPK3-kinase dead (KD))^1^ in A375, EP or IGR cells resulted in spontaneous MLKL phosphorylation (Figure 3a), indicating that reconstitution of functional RIPK3 is sufficient to phosphorylate a known downstream target. We next analysed the respective RIPK3-expressing melanomas for DR-induced, caspase-dependent or -independent cell death (Figures 3b and c and Supplementary Figures 1A and C). Only RIPK3 increasingly promoted CD95L-mediated cell death in A375 cells (Figure 3b) and to a lesser extent, in IGR cells (Supplementary Figure 2A), indicating that active RIPK3 participates in apoptosis regulation as recently demonstrated.^24, 25^ Moreover, the sensitivity to CD95L-mediated cell death was further increased whenever IAPs were inhibited (Figures 3b and c and Supplementary Figure 2A and C), in line with the data in the parental cell lines (Figure 1b). However, whenever caspase activity was inhibited (zVAD-fmk), both CD95L and IAP antagonist/CD95L treatment exclusively unmasked necroptosis in cells expressing functional RIPK3 (Figures 3b and c and Supplementary Figure 2C). These results support the conclusion that RIPK3 protein and its kinase activity are not only involved in regulation of apoptosis but also required for necroptosis execution in melanoma. We further observed an altered cell death morphology in melanoma cells with functional RIPK3, whenever caspases are blocked (Figure 3d). In the presence or absence of cIAPs, cell death exhibited apoptotic morphology on CD95L stimulation, including membrane blebbing, independent of RIPK3 expression. In contrast, another rounded cellular morphology together with a swollen cytoplasm, disintegrated nuclei (HOECHST and SYTOX Green positivity) was detected in the presence of zVAD-fmk indicative of necroptosis (Figure 3d). In summary, reconstitution of functional RIPK3 in melanoma cell lines increasingly promotes DL-induced apoptosis and unmasked DL-induced necroptosis in the absence of caspase and IAP activity. Therefore, functional RIPK3 is necessary for apoptosis and necroptosis execution in malignant melanoma.

IAP antagonists can activate autocrine TNF production in a subset of tumour cells.^26, 27, 28, 29^ We successfully inhibited TNF-mediated apoptosis in previous studies^5^ as well as our control conditions (Supplementary Figure 3A–D) by recombinant TNFR2-Fc. However, CD95L-induced cell death in RIPK3-expressing melanoma cells or their respective controls was unaltered by addition of TNFR2-Fc. This supports that neither apoptosis nor necroptosis in RIPK3-expressing A375 and IGR melanoma cells is dependent on autocrine TNF signalling.

### RIPK3 activates spontaneous and cIAP-protected MLKL phosphorylation

**Figure 4:**
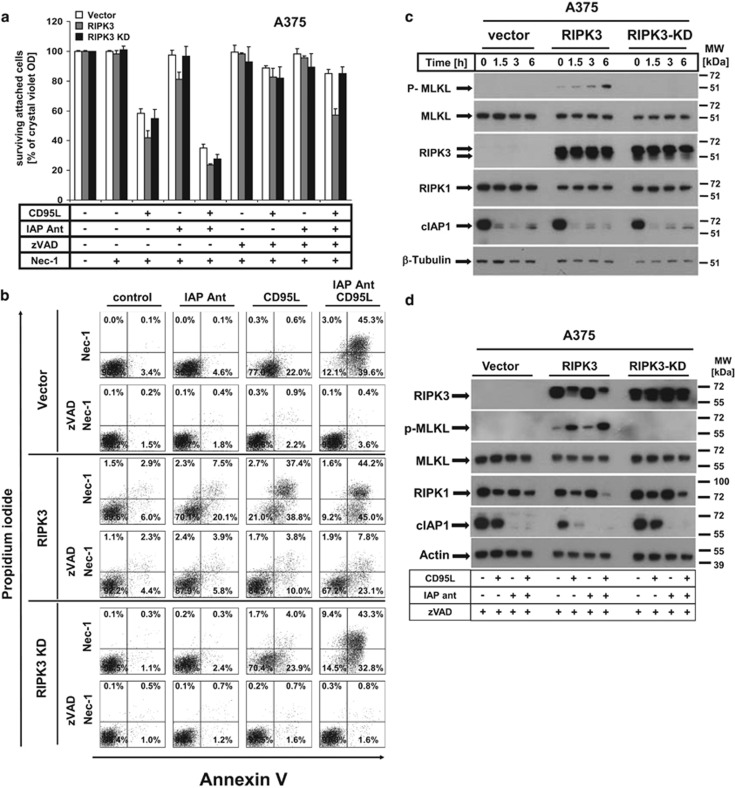
CD95L/IAP antagonist-induced necroptosis in RIPK3-re-expressing A375 cells is partially RIPK1 kinase independent and promotes MLKL phosphorylation. (**a**–**c**) CD95L/IAP antagonist-mediated necroptosis but not CD95L-induced apoptosis in RIPK3-expressing A375 cells is partially RIPK1. (**a** and **b**) Control, RIPK3-, and RIPK3-KD-expressing A375 cells were either pre-stimulated with Nec-1 (50 *μ*M), zVAD-fmk (10 *μ*M), or IAP antagonist (100 nM) alone or in respective combination for 1 h before stimulation or costimulation with CD95L (0.5 U/ml) for 18–24 h (**a**), for 14 h (**b**), followed by analysis with respective methods. (**a**) Crystal violet staining was performed as previously described. Summary of multiple independently performed experiments (four experiments) including the S.E.M. of all experiments is depicted. (**b**) Externalization of phosphatidylserine (PS) and cell death was analysed after Annexin V/PI double staining by FACS analysis as described in Materials and Methods section. One of two independent experiments is shown representatively. For analysis of MLKL phosphorylation, vector control, RIPK3-, and RIPK3-KD-expressing A375 cells were stimulated with IAP antagonist (1 h prestimulation with 100 nM) followed by CD95L (0.5 U/ml) stimulation for the indicated time (**c**). The respective cell lines were either separately pre-stimulated or co-stimulated with IAP antagonist (1 h pre-stimulation with 100 nM) and zVAD-fmk (1 h pre-stimulation with 10 *μ*M), followed by CD95L treatment (0.5 U/ml) for 6 h (**d**). Phosphorylation of MLKL as well as expression of MLKL, RIPK3, RIPK1, cIAP1, actin, and *β*-tubulin was analysed by western blot analysis as previously described. One of three (**c**) and one of two (**d**) representative and independently performed experiments is shown

For the execution of necroptosis, RIPK3-mediated phosphorylation of MLKL requires the interaction of kinase-active RIPK1 with RIPK3 (for review, see Vanden Berghe *et al.*^7^), but RIPK3 overexpression is also able to promote necroptosis independent from RIPK1 activity.^25, 30^ To further elaborate the role of RIPK1/RIPK3 activities in necroptosis execution, we analysed cell death responses of RIPK3-expressing melanoma cells in the presence of the RIPK1 inhibitor Necrostatin-1 (Nec-1) in more detail.^31^ Inhibition of RIPK1 alone did not alter CD95L-induced cell death when IAPs were suppressed irrespective of the level of RIPK3 expression in melanoma cells, indicative of dual activation of necroptosis and apoptosis^5^ (Figures 4a and b). As demonstrated for TNF-mediated necroptosis,^30^ RIPK3-expressing A375 (Figures 4a and b) or, to a lesser extent, IGR melanoma cells (Supplementary Figure 2B and C, lower result panel), but not control transduced melanoma cells, were not fully protected from IAP antagonist/CD95L treatment by ZVAD-fmk and Nec-1 (Figure 4a). The inability of Nec-1 to fully suppress IAP antagonist/CD95L-mediated necroptosis raised the possibility of a potential activation of the necroptotic signalling machinery downstream of RIPK1, as observed in other studies.^30, 32^ Therefore, we next studied the kinetics and extent of MLKL phosphorylation in the presence or the absence of cIAPs (Figures 4c and d) under conditions of necroptosis induction (zVAD/CD95L *versus* zVAD/IAP-antagonist/CD95L) in RIPK3-reconstituted melanoma cells. MLKL phosphorylation was detected in a time-dependent manner within 90 min, with further increase up to 6 h after stimulation in RIPK3-expressing, but not in RIPK3-KD or vector control melanoma cells (Figure 4c). Suppression of cIAPs by IAP antagonist also resulted in an increase in MLKL phosphorylation in RIPK3-reconstituted cells (Figure 4d). These experiments suggested that MLKL phosphorylation indeed not only occurs in a strict RIPK3-dependent manner but is also a consequence of DL stimulation with further increase on cIAPs depletion. Of interest, CD95L stimulation led to a marked shift of the RIPK3-specific signals to a slightly higher molecular weight, indicative of posttranslational modification. This shift may likely be explained by autophosphorylation of RIPK3 on CD95L stimulation.

### CD95L-induced MLKL phosphorylation and necroptosis depends on RIPK1 and RIPK3 kinase activity

**Figure 5:**
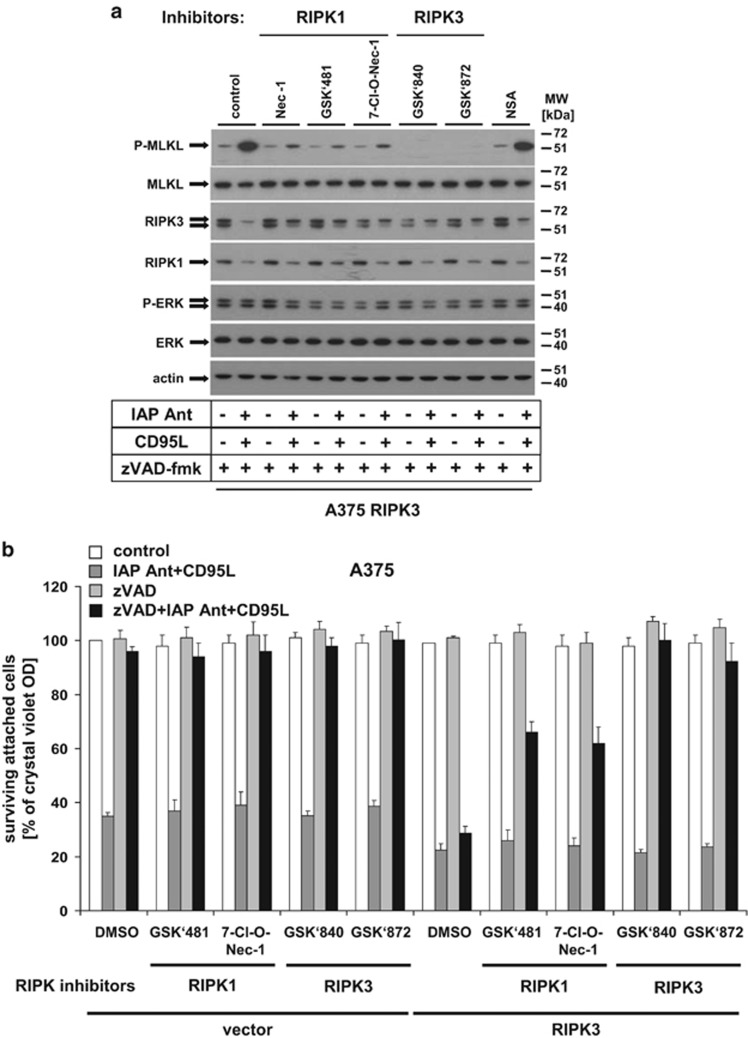
CD95L/zVAD/IAP antagonist-mediated necroptosis induction and MLKL phosphorylation depends on RIPK1 and RIPK3. (**a**) MLKL phosphorylation in RIPK3 expressing cells depends on RIPK1 and RIPK3. RIPK3-overexpressing A375 melanomas were either pre-treated with IAP antagonist (100 nM), zVAD-fmk (10 *μ*M), Nec-1 (50 *μ*M), NSA (10 *μ*M), RIPK3 kinase inhibitor (GSK'840 and GSK'872) or RIPK1 inhibitors (7-Cl-O-Nec-1 and GSK'481; 10 *μ*M each) alone or in respective indicated combinations for 1 h followed by CD95L (0.5 U/ml) costimulation for 6 h. Phosphorylation of MLKL or ERK as well as expression of MLKL, RIPK3, RIPK1, ERK, and actin was analysed by western blot analysis as previously described. One of three representative independently performed experiments is shown. (**b**) RIPK3 inhibitors fully protect RIPK3 reconstituted melanomas against CD95L/IAP antagonist-mediated necroptosis. Vector control and RIPK3-expressing cells were either pretreated with IAP antagonist (100 nM), zVAD-fmk (10 *μ*M), RIPK3 kinase inhibitor (GSK'840 and GSK'872), or RIPK1 inhibitors (7-Cl-O-Nec-1 and GSK'481) alone or in respective combinations followed by CD95L (0.5 U/ml) costimulation for 18–24 h followed by analysis with crystal violet assay as described previously. Summary of multiple independently performed experiments (three experiments) is shown including the S.E.M. of the whole set of experiments

Given the intricate balance of RIPK1 and RIPK3, and their functions as scaffold molecules or kinases, respectively, we next investigated the impact of recently reported chemical inhibitors of RIPK1 and RIPK3 in more detail^24, 33^ (Figure 5a). Spontaneous MLKL phosphorylation mediated by RIPK3 overexpression (Figure 4c) is fully suppressed by RIPK3 inhibitors (GSK'840 and GSK'872), but not inhibited by RIPK1 inhibitors (7-Cl-O-Nec-1 and GSK'481A)^33, 34^ or Nec-1 (Figure 5a). Our findings led us to conclude that RIPK3 overexpression can promote DL-induced necroptosis independently from RIPK1 activity as previously demonstrated.^25, 30^ In contrast, IAP antagonist/zVAD/CD95L-induced MLKL phosphorylation in RIPK3-expressing melanomas was partially suppressed by Nec-1 and other RIPK1 inhibitors but fully suppressed by any of the used RIPK3 inhibitors. RIPK3 inhibition and MLKL phosphorylation correlated with full inhibition of necroptosis (Figure 5b). Furthermore, the lack of complete necroptosis protection by Nec-1 (Figures 4a and b) also correlated with at best partial suppression of MLKL phosphorylation (Figure 5a). Our experiments show that both RIPK3-mediated spontaneous and DL/IAP antagonist-induced MLKL phosphorylation and subsequent necroptosis induction require RIPK3 activity. In contrast, RIPK1 activity is critical for DL-induced, but not for RIPK3-initiated spontaneous MLKL phosphorylation. However, as our experiments show, the previously published inhibitor of MLKL-mediated necroptosis (NSA, necrosulfonamide)^11^ was unable to suppress MLKL phosphorylation (Figure 5a, NSA treatment), and necroptosis inhibition (data not shown) in our cellular models. In summary, our data show that spontaneous MLKL phosphorylation is RIPK3 dependent but is not associated with spontaneous necroptosis induction, indicating that Phospho-MLKL under those conditions either accumulates in an inactive form that is not able to translocate into cellular membranes. Alternatively, there may exist other proteins that bind to and block MLKL translocation and necroptosis, as suggested.^14^ As an additional alternate explanation, additional triggers may be required for MLKL phosphorylation, membrane translocation and finally necroptosis execution.

### Dabrafenib, but not Vemurafenib, interferes with MLKL phosphorylation and necroptosis signalling in RIPK3-expressing melanoma

**Figure 6:**
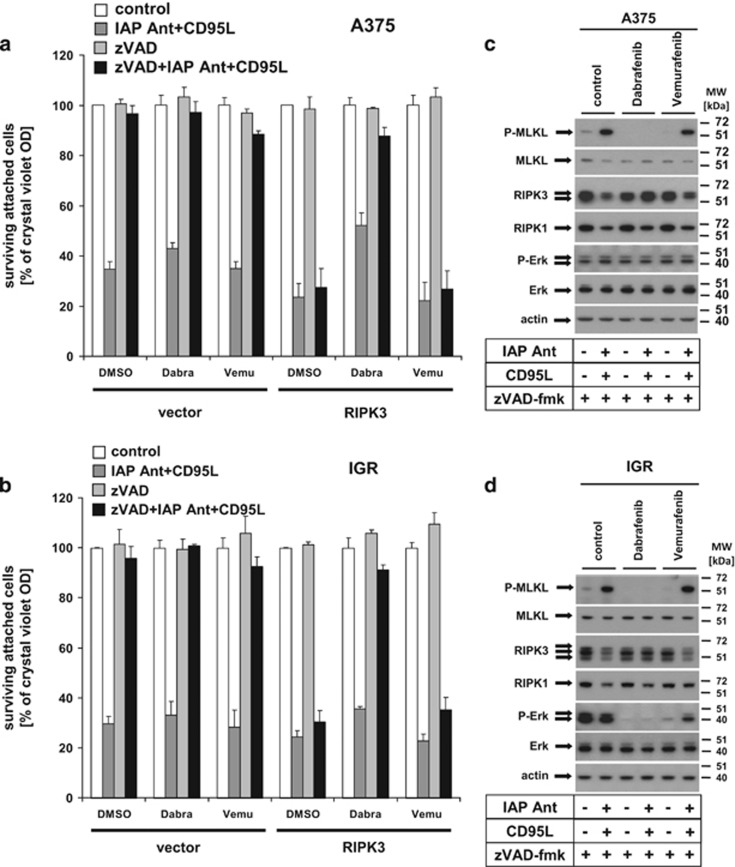
Dabrafenib, but not Vemurafenib, suppresses DL/IAP antagonist-mediated necroptosis by inhibition of MLKL phosphorylation. (**a** and **b**) Dabrafenib blocks necroptosis in RIPK3-reconstituted melanomas. Vector control and RIPK3-expressing A375 (**a**) or IGR (**b**) melanomas were either pretreated for 2 h with IAP antagonist (100 nM), zVAD-fmk (10 *μ*M), Dabrafenib (10 *μ*M), or Vemurafenib (A375 with 30 *μ*M and IGR with 10 *μ*M) alone or in respective combinations followed by CD95L (0.5 U/ml) costimulation for 18–24 h and analysis with crystal violet assay as described previously. Summary of three independently performed experiments is shown. The S.E.M. of the whole set of experiments is depicted. (**c** and **d**) Dabrafenib but not Vemurafenib inhibits MLKL phosphorylation in RIPK3-reconstituted A375 (**c**) and IGR (**d**) melanomas. RIPK3-expressing A375 (**c**) or IGR (**d**) melanomas were either pretreated for 2 h with IAP antagonist (100 nM), zVAD-fmk (10 *μ*M), Dabrafenib (10 *μ*M), or Vemurafenib (A375 with 30 *μ*M and IGR with 10 *μ*M) alone or in respective combinations followed by CD95L (0.5 U/ml) costimulation for 6 h. Phosphorylation of MLKL or ERK as well as expression of MLKL, RIPK3, RIPK1, ERK, and actin was analysed by western blot analysis as previously described. One of three independently performed experiments is shown as representation of the results

BRAF mutations that result in constitutive cell proliferation are present in roughly 50% of malignant melanoma.^35^ The BRAF inhibitors Vemurafenib or Dabrafenib suppress proliferation of BRAF-mutated melanoma cells,^36^ but surprisingly Dabrafenib effectively suppresses RIPK3 activity as an off-target effect.^37^ Thus, it is intriguing to speculate that the effectiveness of a therapy with BRAF inhibitors could be hampered (or altered) by interference with necroptosis/RIPK3 signalling. We therefore explored conditions of necroptosis induction in the presence of Dabrafenib or Vemurafenib in our model systems. In line with the recent report, Dabrafenib but not Vemurafenib blocked necroptosis (Figures 6a and b; black bars) and MLKL phosphorylation (Figures 6c and d), but also protected from DL/IAP antagonist-mediated apoptosis (Figures 6a and b; grey bars) in RIPK3-reconstituted A375 or IGR melanoma cells. Interestingly, both inhibitors strongly repressed extracellular signal-regulated kinase (ERK) phosphorylation in IGR, but not in A375 cells (Figures 6c and d). Our observations thus indicate that in contrast to Vemurafenib, Dabrafenib is a potent inhibitor of MLKL phosphorylation and consequently protects from necroptosis. This was a more general phenomenon for DR signalling, as Dabrafenib also inhibited TRAIL (Supplementary Figures 4A and B) and TNF-mediated necroptosis (Supplementary Figures 4C and D), in line with a recent report in primary and transformed keratinocytes.^38^ Taken together, the absence of RIPK3 or blockade of RIPK3 activity by the respective inhibitors or the BRAF inhibitor Dabrafenib, but not Vemurafenib, are able to block DL-mediated necroptotic and, to some extent, apoptotic cell death in melanoma.

## Discussion

Targeted therapeutics that suppress melanoma growth such as BRAF inhibitors Vemurafenib or Dabrafenib, or the direct MEK inhibitors Cobimetinib or Tranetinib,^36^ have revolutionized metastatic melanoma therapy and have at last improved patient survival. As these therapeutics lose their effectiveness within months, most likely by activation of alternative MEK signalling,^39^ innovative treatments may require additional direct triggering of alternative tumour-specific cell death pathways.^40^ Inhibition of IAPs is one promising strategy to activate cell death and is currently under clinical investigation in various cancer types (for review, see Wan *et al.*^41^). In keratinocyte skin cancer, we described the inhibitory function of cIAPs for apoptosis and necroptosis.^4, 5^ In the current report we investigated the impact of RIPK1 and RIPK3 kinase for apoptosis and necroptosis in malignant melanoma.

Our analysis of a large number of cultured melanoma cells identified largely uniform expression of XIAP and cIAP1, but not of cIAP2. Thus, IAP antagonists may represent promising compounds to sensitize melanoma cells to cell death triggering, possibly in combination with other targeted therapies or chemotherapeutics (for review, see Obexer *et al.*^18^). We detected a substantially increased sensitivity to CD95L-mediated apoptosis but not necroptosis when IAP activity was suppressed. Thus, XIAP and/or cIAPs protect melanoma cells from DL-induced apoptosis (CD95L) as also found in breast^42^ or pancreatic cancer.^43^ XIAP/cIAPs suppression by macrocyclic XIAP antagonists in melanoma and breast cancer support cell death induction and tumour growth inhibition *in vivo*,^44^ indicative of the indispensable protective role of IAPs in many cancer entities. In our studies, melanomas surprisingly lacked the execution of RIPK1-dependent necroptosis, usually uncovered when caspase function is blocked. A pure apoptotic cell death response has been documented in different tissues of genetic mouse models whenever single genes coding for essential components of the necroptotic cell death machinery are absent, most prominently MLKL or RIPK3.^24, 45, 46, 47, 48^

When we examined the cause for loss of necroptosis, we uncovered the vast absence of RIPK3 protein expression in melanomas when compared with melanocytes and nevus cells. The lack of protein expression was mediated by a lack of RIPK3 mRNA (Figure 2) as previously described in lung cancer^49^ and in colon cancer^50^ or in subtypes of metastatic melanoma.^51^ A more general lack of RIPK3 mRNA can be a result of limited promoter activation controlled by epigenetic DNA modification such as DNA methylation, histone deacetylation^52^ or regulated by tumour-initiating signals such as chronic hypoxia.^50^ Thus, our observation and the correlation between necroptosis protection and low RIPK3 expression in melanoma raises the possibility that progression of malignant melanoma may require silencing of RIPK3-dependent necroptosis, or alternatively other RIPK3-dependent signalling pathways, as suggested.^53^ These different hypotheses await experimental clarification in the future.

Previous investigations demonstrated the indispensable role of RIPK3 in cell death regulation during embryonic development,^54, 55, 56^ stimulation with different ligands of the TNF superfamily,^5, 57^ TLR3 agonists,^4, 58^ during etoposide-mediated stress responses^9^ and on interferon signalling or during virus infections.^7, 59, 60^ In our study, reconstitution of RIPK3 not only increased apoptosis as recently reported^8, 24^ but also allowed reactivation of necroptosis in melanoma whenever IAPs are inhibited. Consistent with our observation that human RIPK3-KD mutant D160N is unable to transmit necroptosis, reconstitution of murine kinase-inactive RIPK3-KD mutants such as D161N^45^ or D143N, or K51A^25^ also fail to induce necroptosis, indicating that the RIPK3 catalytic activity is indispensable for necroptotic cell death.^24^ In contrast to the observed apoptosis induction in murine cells that expressed the RIPK3 kinase-inactive mutant D161N,^24, 45^ reconstitution of human RIPK3-D160N did not interfere with spontaneous apoptosis or led to altered sensitivity to DL or IAP antagonist, indicating that the RIPK3 catalytic activity is dispensable for apoptosis. Thus, our data extend the previous notion that RIPK3 may either be a critical downstream target or a necessary part of intracellular signalling complexes such as the Necrosome or Ripoptosome. If RIPK3 acts as a kinase or may also modulate the stoichiometry of protein complexes by a presumed scaffold function is an intriguing question that needs to be addressed in the future.^24, 61^

Interestingly, RIPK3-expressing melanoma cells, but not RIPK3-KD cells, show spontaneous and increased MLKL phosphorylation (Figure 4c), a strong indication that kinase activity of RIPK3 is the missing link for DL-mediated necroptosis in melanomas. Both spontaneous and IAP antagonist/DL-mediated MLKL phosphorylation following necroptosis induction is blocked in the presence of specific RIPK3 inhibitors.^24^ These data demonstrate (a) the functionality of RIPK3 for activation of its downstream target within the necroptotic signalling machinery and (b) the requirement of the kinase function for this phosphorylation event in melanoma cells. In our previous studies,^5^ we found that RIPK1 was critical for necroptosis execution in SCC cells. In contrast, other cell types with high RIPK3 expression failed to be protected from cell death by interference with RIPK1 activity.^8, 25^ When RIPK1 is inhibited by Nec-1, 7-Cl-O-Nec-1 or GSK'481,^62^ our data also show a partial RIPK1-independent execution of necroptosis. These results are supported by other recent reports that highlight the intricate interplay between RIPK1 and RIPK3, and its dependency from the stoichiometry of the different critical proteins.^24, 25, 30^ Taken together, the cellular context, RIPK1 and RIPK3 expression level, and known (or unknown) downstream molecules such as MLKL intensely have an impact on the cell biological response to identical stimuli. Possibly, other currently unknown signalling pathways can be activated only by RIPK3, which also involves other interacting proteins that triggers RIPK3 phosphorylation. Alternatively, RIPK3 may also assemble in homomultimers and is activated by autophosphorylation. How this influences the tumour response (e.g., activation of immune cells to dying melanoma cells) in a tumour-bearing patient remains to be elucidated in the future.

Reactivation of the RIPK1/RIPK3/MLKL signalling platform by RIPK3 reconstitution can overcome necroptosis resistance in melanoma and therefore could be used as potential death-inducing targeted therapy against melanoma. Our data presented in the current study indicate that a combination therapy of Dabrafenib or Vemurafenib together with reagents that allow RIPK3 reconstitution could be such a strategy that was tested *in vitro* in melanoma (Figure 6). Consistent with another recent study,^37^ Dabrafenib also interfered with RIPK3 activity in our study (Figure 6 and Supplementary Figure 3) and was able to block necroptosis and, to some extent, apoptosis. Based on the observation that RIPK3 also increasingly promotes apoptosis (Figures 3b and c and Cook *et al.*^25^), the described off-target effect of Dabrafenib^37^ for inhibition of RIPK3 activity could be responsible not only for inhibition of necroptosis but also for RIPK3-dependent apoptosis. It is also possible that other currently unknown molecules of the apoptotic signalling machinery are influenced by the off-target activity of Dabrafenib. In contrast, the alternative BRAF inhibitor Vemurafenib was unable to suppress MLKL phosphorylation and necroptosis, indicating that suppression of BRAF/MEK-mediated proliferation and RIPK3-mediated necroptosis are not interconnected. Dabrafenib-mediated necroptosis suppression strongly correlated with its inhibitory effect on MLKL phosphorylation. Although the exact molecular mechanisms how BRAF inhibition and necroptosis protection cross-talk are fully unknown, the surprising discordant response of two different BRAF inhibitors demonstrated in this study merits further attention in the future. For therapeutic intervention, the reactivation of the RIPK1/RIPK3/MLKL necroptotic signalling axis together with simultaneous inhibition of BRAF-mediated proliferation (e.g., Vemurafenib) without effecting RIPK3 activity could be an attractive strategy.

## Material and Methods

### Materials

The following Abs were used for western blot analysis: XIAP (H62120) (Transduction Laboratories, San Diego, CA, USA), rat Abs to cIAP1 (Silke *et al.*^63^) and cIAP2,^22^ anti-*β*-tubulin (clone 2.1) and anti-actin antibodies (Sigma, St Louis, MO, USA), RIPK3 polyclonal antibody (IMGENEX, San Diego, CA, USA), RIPK1 (Transduction Laboratories), anti-MLKL (phospho-S358) antibody (Abcam, Cambridge, UK), and anti-ERK-2 and anti-phospho-ERK from (Santa Cruz, Dallas, TX, USA). MLKL antibodies recognizing both mouse and human MLKL^46^ were kindly provided by James Murphy (WEHI, Melbourne, Parkville, Australia). Horseradish peroxidase (HRP)-conjugated goat anti-rabbit, goat anti-rat IgG, and goat anti-mouse IgG Abs, and HRP-conjugated goat anti-mouse IgG1, IgG2a, and IgG2b were obtained from Southern Biotechnology Associates (Birmingham, AL, USA).

### Cell culture

The human melanoma cell lines PM-WK, RPM-MC, RPM-EP, MM-RU, MM-AN, and MM-LH were kindly provided by Randy H Byers (Department of Dermatology, Boston University School of Medicine) and cultured as previously described.^64^ The following human melanoma cells were obtained either from ATCC (Manassas, VA, USA) or DSMZ (Deutsche Sammlung von Mikroorganismen und Zellkulturen GmbH, Braunschweig, Germany) and cultured as described: A375 (ATCC CRL-1619), MeWo (ATCC HTB-65), SK-Mel-30 (SK-Mel; ACC 151), and IGR-37 (IGR; ACC 237). PREYER melanoma cells (generated from a subcutaneous melanoma metastasis) were kindly provided by A Schwaaf and EB Bröcker. Melanoma cells were cultured in complete DMEM medium (Gibco, Life Technologies, Darmstadt, Germany) with 4 mM L-glutamine adjusted to contain 4.5 g/l glucose, 10% fetal bovine serum (FCS Gold, PAA, Cölbe, Germany), 1% sodium pyruvate, and HEPES buffer. Primary human melanocytes (Mel) and nevus cells were prepared as described^65^ and cultured in complete M2 melanocyte growth medium (PromoCell GmbH, Heidelberg, Germany). HaCaT keratinocytes were kindly provided by Petra Boukamp (DKFZ, Heidelberg, Germany) and cultured as described.^66^

### Substrates

For cell death induction and the analysis of quality and quantity of cell death, the following substrates were used in standard concentrations: ZVAD-fmk (Z-Val-Ala-Asp-fluoromethyl ketone; 10 *μ*M) was purchased from Bachem (Heidelberg, Germany) and NSA (10 *μ*M) was provided by L Sun and X Wang.^11^ Nec-1/Necro-1 (50 *μ*M) was from Sigma and RIPK3 inhibitors (GSK'840 or GSK'872; 10 *μ*M) and RIPK1 inhibitors (GSK'481 or 7-Cl-O-Nec-1/Necro-1) were from Glaxo Smith Kline Corp. (GSK, New York, NY, USA). The IAP antagonist (compound A; 100 nM used in most studies) was generously provided by Tetralogics Corp., Philadelphia, PA, USA. For expression of Fc-CD95L, we used constructs published elsewhere.^67^ One unit of Fc-CD95L was determined as a 1 : 500 dilution of the stock Fc-CD95L supernatant and 1 unit/ml of Fc-CD95L supernatant was sufficient to kill 50% (LD50) of A375 melanoma cells, as described.^68^ PI was obtained from Sigma, crystal violet was from VWR International (Radnor, PA, USA). TNFR2-Fc (Enbrel; Pfizer Inc., New York, NY, USA) was used at a concentration of 10 *μ*g/ml. HF-TNF was prepared as described^68^ and used at a concentration of 10 ng/ml. Vemurafenib (PLX 4032) and Dabrafenib (GSK 2118436) were purchased from Seleckchem (Houston, TX, USA). All RIPK1/RIPK3 inhibitors used, and Vemurafenib and Dabrafenib were tested in preliminary experiments for cell toxicity. The highest non-toxic or low toxic concentration of the respective inhibitors was used in subsequent experiments.

### Retroviral infection

To overexpress RIPK3 or RIPK3-KD (KD mutant, D160N), the respective cDNAs were subcloned from pEGFP N1 vectors into pCFG5-IEGZ retroviral vector by standard cloning procedures and verified by sequencing. Sequence-confirmed vectors were used for transduction of A375, IGR, and EP melanoma cells, respectively. For virus production, the amphotrophic producer cell line ΦNX was transfected with 10 *μ*g of the retroviral vectors by calcium phosphate precipitation. Cell culture supernatants containing viral particles were generated by incubation of producer cells with A375 medium DMEM containing 10% FCS, sodium pyruvate, and HEPES buffer) overnight. Following filtration (45  *μ*M filter, Schleicher & Schuell, Dassel, Germany), culture supernatant was added to the respective melanoma cells seeded in six-well plates 24 h earlier in the presence of 1 *μ*g/ml polybrene. After centrifugation for 2 h at 30 °C, viral particle containing supernatants were replaced by fresh medium. After 14 days of zeocine selection of bulk infected A375, IGR, and EP cell cultures, fluorescence-activated cell sorting (FACS) analysis for green fluorescence protein expression (always >95%, data not shown) and western blot analysis were performed on polyclonal cells to confirm ectopic expression of the respective molecules. The empty retroviral vectors served as controls. Aliquots of cells were used for the experiments between passages 2–6 after initial characterization for all subsequent studies. HaCaT cells expressing IKK2-KD and vector control were established to control for effectivity of TNF-R2-Fc for TNF-mediated apoptosis as previously described.^22^

### Western blot analysis

Cell lysates were prepared as described^69^ and 5 *μ*g of total cellular proteins were separated by SDS-PAGE on 4–12% gradient gels (Invitrogen, Karlsruhe, Germany) followed by transfer to nitrocellulose or PVDF membranes. Blocking of membranes and individual incubation with primary and appropriate secondary Abs were performed as recommended by the companies respectively. Bands were visualized with ECL detection kits (Amersham, Freiburg, Germany).

### Immunofluorescence microscopy

For detection of nuclear morphology and integrity of the cell membrane, 5 × 10^4^ A375 cells expressing vector control, RIPK3, and RIPK3-KD were seeded per well in a 12-well plate. Following 24 h of incubation for adherence, cells were stimulated as indicated in the figure legend for 14 h. Subsequently, cells were incubated with Hoechst 33342 (5 *μ*g/ml; Polysciences Europe, Eppelheim, Germany) and SYTOX Green (5pM; Invitrogen, Molecular Probes, Eugene, OR, USA) for 15 min at 37 °C, immediately followed by phase-contrast or fluorescence microscopy using a Leica DMIRB with integrated camera Leica DFC 450C (Leica Microsystems, Wetzlar, Germany). Digital images were identically processed using the advanced LAS version 4.4.0, Build: 454 Leica Microsystems.

### mRNA isolation and qPCR

For quantification of RIPK3 mRNA expression in parental melanomas, primary melanocytes, and nevus cells (Figure 2b), total mRNA was isolated with RNeasy Kit (Qiagen, Hilden, Germany). cDNA was synthesized by SuperScript II Reverse Transcriptase (Invitrogen, Carlsbad, CA, USA). RT qPCR analysis was performed by using KAPA SYBR Fast qPCR (Peqlab, Erlangen, Germany) in the Mx3005P (Stratagene, La Jolla, CA, USA) real-time thermal cycler. Equal cycling conditions were used to amplify genes of interest and reference gene products. HotStarTaq DNA Polymerase was launched by an initial step of 15 min at 95 °C followed by 42 cycles of 1 step (denaturation) at 94 °C for 15 s, 1 step (annealing) at 55 °C for 30 s, and 1 step (extension) at 72 °C for 30 s. Melting curve analysis was used to confirm the specific product amplification. RT-PCR efficiency was calculated using standard curve (plotted as a logarithmic function of the cDNA dilution factor) and Mx3005P software (Stratagene). Normalization was performed with primers to *β*-actin as described.^69^ The following primer sequences were used for qPCR analysis: RIPK3 forward 5′'-CAAGATCGTAAACTCGAAGG -3′, RIPK3 reverse 5′-CCGTTCTCCATGAATTTAGT-3′ *β*-actin forward 5′-AGAAAATCTGGCACCACACC-3′, *β*-actin (ACTB) reverse 5′-GGGGTGTTGAAGGTCTCAAA-3′.

### Cytotoxicity assays

#### Analysis of living attached cell by crystal violet staining

Cells (1 × 10^4^) were seeded per well in a 96-well plate and cultured for adherence overnight. Cells were stimulated with controls (dimethyl sulfoxide (DMSO) and ethanol), IAP antagonist, zVAD-fmk, Nec-1/Necro-1, NSA, RIPK1 inhibitors (GSK‘481 or 7-Cl-O-Nec-1/Necro-1), RIPK3 inhibitors (GSK‘840 or GSK'872), TNFR2-Fc or CD95L alone, or in respective combinations in 96-well plates accordingly described in the respective figure legends. Crystal violet staining of attached, living cells was performed 18–24 h after stimulation in triplicate wells per condition as described elsewhere.^70^ The optical density of control cultures was normalized to 100% and compared with stimulated cells. In case of combined stimulation of inhibitors together with CD95L or TNF (Supplementary Figure 2D), the spontaneous cytotoxic effect of single diluents and substrates was subtracted from each costimulation to solely show specific DL-induced cell death. For statistical analysis, the S.E.M. was determined for three to four independent experiments of each cell line and stimulatory condition.

#### Annexin V externalization

For detection of phosphatidylserine externalization, cells were stimulated as indicated in the figure legends. Fourteen hours after incubation of cells, trypsinized cells, and the supernatants were collected and resuspended in 1 × Annexin-V binding buffer (10 mM Hepes, pH 7.4, 140 mM NaCl, 2,5 mM CaCl_2_) and 2–4 × 10^5^ cells were subsequently stained with Cy5-conjugated Annexin-V exactly according to the manufacturer's instructions (Pharmingen, San Diego, CA, USA), followed by counterstaining (PI; 10 *μ*g/ml) for 15 min in the dark at room temperature. For all experiments, 1 × 10^4^ cells were analysed by FACScan (Becton Dickinson & Co., San Jose, CA, USA) and summarized with FCS Express version 3 programs (*De Novo* Software, Glendale, CA, USA). Non-stimulated cells served as negative control for Annexin V/PI double stainings.

### Analysis of CD95 surface expression

For analysis of CD95 cell surface expression from vector control and RIPK3-overexpressing A375 and IGR cells on stimulation with control (DMSO) and Dabrafenib (10  *μ*M) for 2 h were stained with anti-CD95 (Apo-1 IgG1) primary antibody as well as isotype-matched control antibodies followed by FACS analysis as described in detail in Diessenbacher *et al.*^22^

## Abbreviations

CD95: cluster of differentiation 95
cIAP1/2: cellular inhibitor of apoptosis protein-1/2
DL: death ligand
DMSO: dimethyl sulfoxide
DR: death receptor
ERK: extracellular signal-regulated kinase
FACS: fluorescence-activated cell sorting
IAP: inhibitor of apoptosis protein
KD: kinase dead
MEK: mitogen-activated protein/extracellular signal-regulated kinase kinase
MLKL: mixed-lineage kinase domain-like protein
NSA: necrosulfonamide
Nec-1/Necro-1: necrostatin-1
PI: propidium iodide
RIPK1: receptor-interacting protein kinase-1
RIPK3: receptor-interacting protein kinase-3
TRAIL: TNF-related apoptosis-inducing ligand
TNF: tumour necrosis factor
TNFR2: tumour necrosis factor receptor-2
XIAP: X-linked IAP
