# Supplementary figures and images for: Absence of RIPK3 predicts necroptosis resistance in malignant melanoma
Source: Cell Death Dis. 2015 Sep 10;6(9):e1884–. doi: 10.1038/cddis.2015.240 (PMC4650439; doi:10.1038/cddis.2015.240)

A

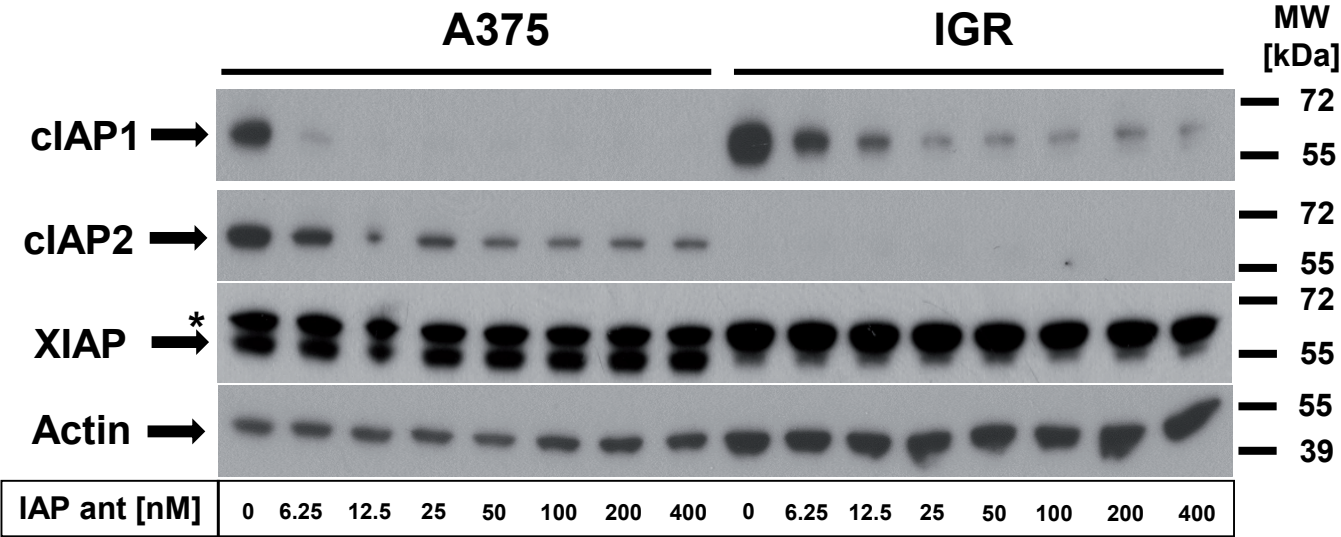

B

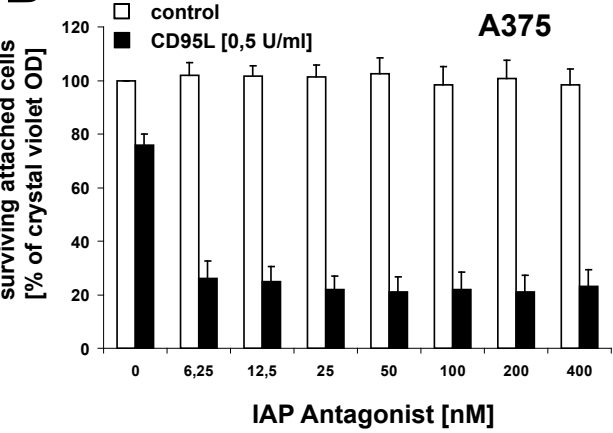

C

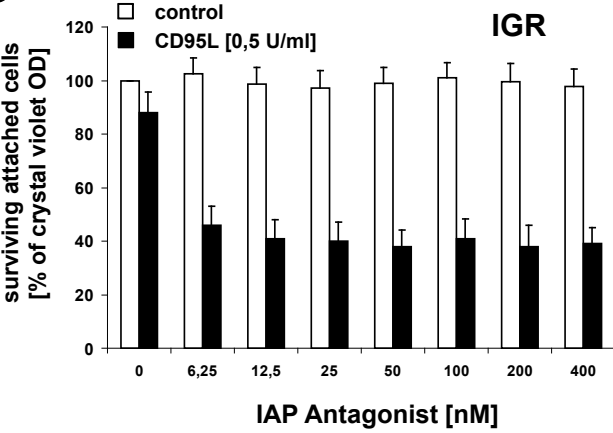





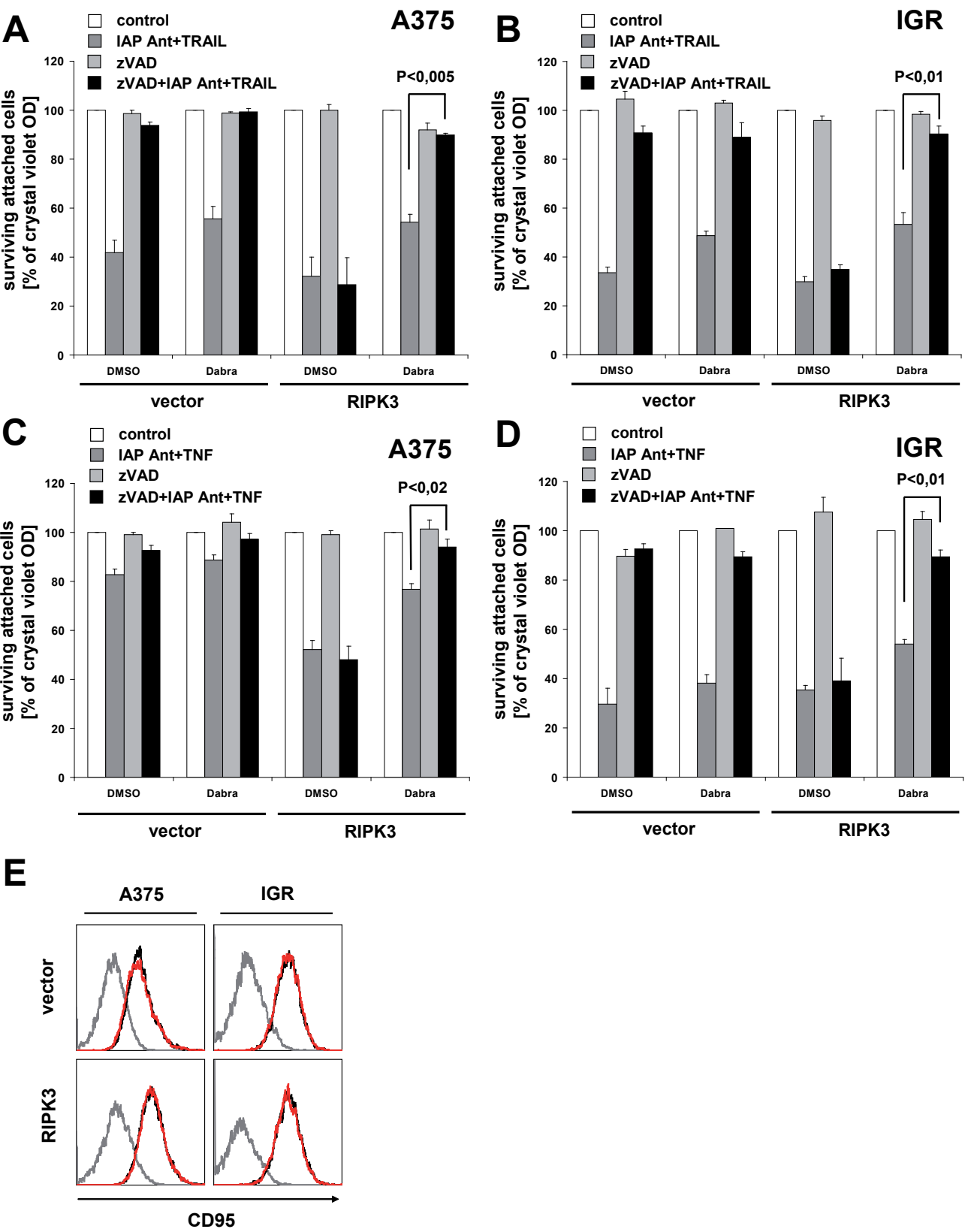

Geserick et al., Supplementary figure 4

Supplement: Supplementary Figures [file cddis2015240x1.pdf]
