# Supplementary material for: Absence of RIPK3 predicts necroptosis resistance in malignant melanoma
Source: Cell Death Dis. 2015 Sep 10;6(9):e1884–. doi: 10.1038/cddis.2015.240 (PMC4650439; doi:10.1038/cddis.2015.240)
Supplement: Supplementary Figure Legends [file cddis2015240x2.doc]

**Supplementary Figures**

**Supplementary Figure 1. IAP antagonist promotes cIAP degradation in a dose-dependent manner and promotes CD95L-induced apoptosis by suppression of XIAP rather than by cIAP degradation.** A. A375 and IGR cells were stimulated with the indicated concentrations of IAP antagonist. Expression of cIAP1, cIAP2, XIAP (asterisk denotes an unspecific band recognized by the utilized XIAP antibody), and β-tubulin (loading control) was determined by Western blot analysis. B. and C. A375 and IGR cells were pre-stimulated with indicated concentration of IAP antagonist alone for 1h followed by stimulation or costimulation with CD95L (0,5U/ml) for 18-24 hrs. Surviving attached cells were quantified with crystal violet assay as described in material and methods. Summary of two independent experiments is shown. The SEM of the whole set of experiments is depicted.

**Supplementary Figure 2. RIPK3 reconstitution in IGR cells promotes zVAD/IAP antagonist/CD95L mediated necroptosis in a RIPK1-dependent manner.** A.-C. A375 cells with expression of vector control, RIPK3 and RIPK3-KD were either non-stimulated or pre-stimulated with zVAD-fmk (10µM), Necrostatin-1 (50µM) or IAP antagonist (100 nM) alone or in combination for 1h before stimulation or costimulation with CD95L (0,5U/ml) for 18-24 hrs. A.-B. Surviving attached cells were quantified with crystal violet assay as described in material and methods section. Summary of three independently performed experiments is shown. Error bars show the SEM of the whole set of experiments. C. For phosphatidyl serine (PS) externalization and cell death quantification, Annexin V/PI double staining was performed. A375 cells with expression of control (vector), RIPK3 and RIPK3-KD were pre-stimulated with DMSO/Ethanol (control), zVAD-fmk (10µM) or IAP antagonist (100 nM) alone or in respective combination for 1h before stimulation or costimulation with CD95L (0,5U/ml) for 18-24h (A. and B.) or for 14h (C) followed by analysis with respective methods. A. and B. Crystal violet staining was performed as previously described. Summary of multiple independently performed experiments (three experiments) is shown. Error bars indicate SEM of the whole set of experiments. C. Externalization of PS and cell death were analysed after Annexin V/PI double staining by FACS analysis as described in material and methods. One of two representative, independently performed experiments is shown.

**Supplementary Figure 3. Apoptotic and necroptotic cell death in RIPK3 reconstituted melanomas is independent from TNF signalling.** A.-C. A375 cells (A. and C.) or IGR cells (B) with expression of vector control or RIPK3 were either non-stimulated or pre-stimulated with zVAD-fmk (10µM), TNF-R2-Fc (10 mg/ml) or IAP antagonist (100 nM) alone or in respective combination for 1h before stimulation or costimulation with CD95L (0,5U/ml) for 18-24 hrs (A. and B.) or 14h (C) followed by analysis with respective methods. A.-B. Surviving attached cells were quantified with crystal violet assay as described in material and methods section. Summary of two independently performed experiments is shown. The SEM of the whole set of experiments is depicted. C. Externalization of PS and cell death were analysed after Annexin V/PI double staining by FACS analysis as described in material and methods section. One representative of totally of two independent performed experiments is shown. D. TNF-R2-Fc sufficiently blocks TNF-mediated apoptosis in IKK2-KD HaCaT cells. Transduced HaCaT cells with expression of vector control and IKK2KD were either non-stimulated or pre-stimulated with TNF-R2-Fc (10 µg/ml) for 1h followed by costimulation with 10 ng/ml recombinant TNF for 18-24 hrs. Surviving attached cells were quantified with crystal violet assay. Summary of two independently performed experiments is shown. Error bars depict the SEM of both experiments.

**Supplementary Figure 4. Dabrafenib suppresses TRAIL and TNF-induced necroptosis in melanomas with RIPK3 expression.**

A – D. Dabrafenib blocks TRAIL- and TNF-mediated necroptosis in RIPK3 reconstituted melanomas. Vector control and RIPK3 expressing A375 (A and C) or IGR (B and D) melanomas were either pretreated for 2h with DMSO (control/ethanol), IAP antagonist (100 nM), zVAD-fmk (10 µM) or Dabrafenib (A375 with 30 µM; IGR with 10 µM) alone or in respective combinations followed by TRAIL (A375: 200 ng/ml; IGR: 100 ng/ml; A and B) or TNF (500 ng/ml; C and D) costimulation for 18-24h and analysis with crystal violet assay as described previously. Summary of three independently performed experiments is shown including the SEM (error bars) of the three experiments. SEM and significance as outlined in material and methods are depicted. E. CD95 cell surface expression in Melanomas is not affected by Dabrafenib. Vector control and RIPK3 expressing A375 or IGR melanomas were treated with DMSO (control/ethanol; black curves) or Dabrafenib (A375 with 30 µM; IGR with 10 µM; red curves) for 2h. Respective cells were stained with anti-CD95 (Apo-1 IgG1) primary antibody as well as isotype matched control antibodies (gray curves) followed by FACS analysis. One of two representative and independent experiments is shown.
